# Spatial-Temporal Evolution and Influencing Factors of Urban Green and Smart Development Level in China: Evidence from 232 Prefecture-Level Cities

**DOI:** 10.3390/ijerph19073939

**Published:** 2022-03-25

**Authors:** Lingyan Xu, Dandan Wang, Jianguo Du

**Affiliations:** 1 School of Management, Jiangsu University, Zhenjiang 212013, China; 2211910022@stmail.ujs.edu.cn (D.W.); jgdu2005@163.com (J.D.); 2 Research Center for Green Development and Environmental Governance, Jiangsu University, Zhenjiang 212013, China

**Keywords:** urban GSDL, spatial-temporal evolutionary heterogeneity, quantile regression tests, threshold effect

## Abstract

Green and smart city is an optimal choice for cities to realize their modernization of governance capacity and sustainable development. As such, it is necessary to clarify the evolutionary characteristics and driving mechanism of urban green and smart development level (GSDL) systematically. From the perspective of green total factor productivity (GTFP), this study adopted the SBM-GML (slack-based model & global Malmquist–Luenberger) method to measure the urban GSDL considering smart input-output elements. Based on the panel data of China’s 232 prefecture-level cities from 2005 to 2018, the spatial and temporal evolution characteristics of urban GSDL were explored, and the factors and structural mutation points affecting urban GSDL were analyzed with quantile regression tests and threshold regression tests. The findings of this paper showed that (1) there is an upward trend in the volatility of urban GSDL from 2005 to 2018, in which the eastern region was highest, followed by the central and western regions, and the differentiation showed no converge among regions; (2) the effect of technical progress and technical efficiency improvement on the urban GSDL was demonstrated with a fluctuating “Two-Wheel-Drive” trend on the whole; (3) the urban GSDL was promoted by the opening-up level and urban scale significantly, while inhibited by the level of economic development and government size. Additionally, the effects of industrial structure, financial development level, and human capital level on the urban GSDL were distinctive at different loci; (4) the threshold effects of economic and financial development level on improving the positive effects of industrial structure and opening-up level on urban GSDL were significant. These findings may enrich the research literature on the evolutionary heterogeneity of green and smart cities and provide theoretical and practical exploration for the construction of green and smart cities.

## 1. Introduction

China’s urbanization level reached 63.89% by the end of 2021 [1], and it has been demonstrated that the development of industrialization and urbanization contributed significantly to China’s rapid growth of the economy [2]. However, the deterioration of ecology, frequent extreme weather, and other environmental problems caused by urbanization also became very serious [3]. Thus, China has successively constructed low-carbon pilot cities and smart cities since 2010, then the green and smart cities have been built based on green, low-carbon, and sustainable development [4,5]. The construction of green and smart cities relies on the coordinated development of green and smart elements, in which green development is the internal requirement for constructing the smart city, and smart elements provide a digitized mode for urban green development. Eventually, they are mutually integrated to promote each other. It is worth noting that the resource endowment of cities in China is diverse, there is heterogeneity in technology innovation and economic development. As such, the urban green and smart development level (GSDL) would vary considerably across the country [6]. Therefore, the urban GSDL should be developed reasonably with more consideration of resources, environmental elements, and smart elements from the perspective of urban input-output resources. It will have a profound impact to verify the spatial and temporal evolution trend of the urban GSDL in China more systematically and scientifically.

Given the evaluation related to the urban GSDL, the total factor productivity (TFP) is extensively adopted [7], which focuses on the economic development considering input-output indicators. Then, energy consumption and undesirable pollution emission are further considered to improve the calculation of TFP [8,9]. Since then, the efficiency of urban green development [10,11], logistics industry efficiency [12], and agricultural green development efficiency [13] has been explored from different levels based on TFP. Furthermore, the green total factor productivity (GTFP) is conceptualized and standardized by considering the capital and environmental constraints to measure the quality of economic growth [14] and the data envelopment analysis (DEA) is primary to be adopted to measure the GTFP [15,16]. In addition, the comprehensive evaluation method conducted from multi-dimensional perspectives has been widely adopted to evaluate urban green development as well as smart development [17,18]. Moreover, the combination of the PCA-GRA, entropy weight, and cloud model are also adopted to explore the evolution of the urban GSDL [19].

Distinct heterogeneities related to urban GSDL have focused on how the urban GSDL evolves from both spatial and temporal dimensions. Liu [20] verified the evolution trend of GTFP in differential industrial enterprises caused by the industry heterogeneity based on the global SBM model combined with the SYS-GMM regression model. Further, the spatial relations have been adopted into the deepening analysis [1,12], and then the spillover effect of regional GTFP on the surrounding areas are revealed, thus some countermeasures and suggestions to promote the quality of urban development from the spatial perspective are proposed [21]. Related to the urban green development, Wang [22] explored the PM2.5 distributions and relevant key drivers in major China by considering both spatial and temporal heterogeneities. Tian [23] adopted the undesirable SBM model to measure the green innovation efficiency of 286 prefecture-level cities from 2005 to 2017, and the results showed that the overall urban green innovation efficiency was low, and manifested as the national fluctuation, eastern transition, central collapse, and northeast stagnation.

In terms of the influencing factors of the urban GSDL, literature has focused on the identification and quantification of factors that drive urban green and smart development in China. The construction of green and smart cities would be affected by various factors under the constraints of limited resources and environmental factors, in which the regional development level is regarded as the main factor [24]. The development of the economic level is conducive to promoting the agglomeration of superior resources and improving the urban innovation level [15]. Moreover, the driving force of smart cities in accelerating economic growth is enhanced steadily with the improvement of financial development and information sharing [25]. The influence of smart factors on the efficiency and quality of urban development has been furtherly analyzed [26], among which the urban innovation level, the degree of government intervention, financial development level [27,28], foreign trade [29], and urbanization have been demonstrated to be conducive to the green economic development, industrial structure [30], and modernization [31,32]. Additionally, telecommunication construction is supposed to break regional market restrictions and reduce the coordination cost of enterprises, which is crucial to facilitate integrated market construction and improve production efficiency through the scale and intensive economy [33]. As the intangible facilities of the city, the human capital is regarded as the crucial element for the urban GTFP [34,35]. Therefore, it can be concluded that both green factors and smart factors show positive effects on urban development. However, the construction of green and smart cities needs long-term accumulation and investments, which might need to reach a threshold to achieve sustainable growth. It has been found that the higher the innovation level of high-technical industries is, the better the smart construction would be [36,37]. Moreover, it has been demonstrated that there are threshold effects of economic development, infrastructure construction, financial development, and technological innovation on GTFP in countries along with the “Belt and Road” [38,39].

According to the above analysis, the GTFP has been further extended from various perspectives, and many influencing factors of urban GSDL such as technological innovation, human capital level, and other factors have been explored, which provide a significant basis for this study. However, previous research attaches less importance to the relationship between green and smart inputs and outputs in modern urban construction; neither take into consideration combining green and smart factors into a unified analytical framework. Furthermore, previous research related to the influencing factors and threshold effects on the urban green and smart development is insufficient, which is not conducive to identifying the key elements of green and smart city construction in the future. Thus, this paper contributes to enriching the research to evaluate the temporal and spatial evolutionary heterogeneity of the urban GSDL scientifically and systematically in China by adopting a more comprehensive measurement including green and smart factors as input and output elements. Based on the evaluation of urban GSDL, this paper aims to explore the impacts of industrial structure, opening-up level, and other control variables on the urban GSDL. Then, the threshold effects will be analyzed to provide meaningful proposals for the high-quality construction of green and smart cities from the aspects of economic and financial development level.

## 2. Materials and Methods

### 2.1. Data Source

This paper collected 232 prefecture-level cities in China as research samples, and the data were obtained from trustworthy channels to ensure the credibility of the research. Given the integrity and continuity of panel data, the samples with serious missing data were not included, and then 232 cities were obtained, among which a small number of missing data were obtained by the linear interpolation method. The data were primarily based on the China Urban Statistics Yearbook, China Statistical Yearbook, China Science and Technology Yearbook (2006–2019), and the Statistical yearbooks. Statistical bulletins of each province and city from 2005 to 2018 were referenced.

### 2.2. The Evaluation of the Urban GSDL

#### 2.2.1. SBM Model Considering Undesirable Output

DEA (data envelopment analysis) is adopted to evaluate the urban GSDL in this paper, which estimates the efficiency of DMU (decision-making unit) from the perspective of input-output, where DMU represents each city. If there is non-zero slack in inputs or outputs, the DEA efficiency calculation based on angle and radial direction will result in deviation of efficiency estimation due to the factor neglection or overestimation. As such, Tone [40] developed the angle of directional distance and put forward the improved DEA model based on slack variables, which is named the SBM model, then the estimation bias caused by radial and angle selection differences was eliminated. Thus, the SBM model was demonstrated to be suitable for evaluating the efficiency of decision-making units with multiple inputs and outputs has widely applied in the measurement of industrial and urban GTFP [41,42]. The SBM model containing the undesirable outputs is as follows:

(1) $$\min\rho =\frac{1-\frac{1}{m}\sum_{i=1}^{m}s_{i}^{-}/x_{i0}}{1+\frac{1}{s_{1}+s_{2}}(\sum_{i=1}^{s_{1}}s_{r}^{g}/y_{r0}^{g}+\sum_{i=1}^{s_{2}}s_{r}^{b}/z_{r0}^{g})},$$

(2) $$s.t.\;X\lambda +s_{i}^{-}=x_{k},\;Y^{g}\lambda -s_{r}^{g}=y_{0}^{g},\;Z^{b}\lambda +s_{r}^{b}=z_{0}^{b},$$

(3) $$\lambda ,\;s_{i}^{-},\;s_{r}^{g},\;s_{r}^{b}\geq 0.$$

where *ρ* represents the efficiency value of cities. To estimate the efficiency of DMU, min *ρ* is adopted in the formulation. *m*, *s*_1_, *s*_2_ represents the vectors of inputs, desired, and undesired outputs respectively, and *s^−^*, *s^g^*, *s^b^* are the corresponding slack vectors. To be specific, *s^−^* represents the redundancy of input *i*, *s^g^* represents the deficiency of desired output *r*, *s^b^* represents the deficiency of undesired output and *λ* is the adjustment matrix. *X* represents the input at the front edge, and *Y* represents the output at the front edge. The range of *ρ* ∈ [0, 1], and only when *s^−^* = *s^g^* = *s^b^* = 0, *ρ* = 1 can be achieved, which indicates that the decision-making units are efficient. When *ρ* < 1, the decision-making units are non-efficient. Suppose that *X* ≥ 0. If *x_io_* = 0, then $s_{i}^{-}/x_{i0}$ will be deleted from the target function. If *y* ≤ 0, it will be replaced by a small positive number. In the objective function of the model, $\frac{1}{m}\sum_{i=1}^{m}s_{i}^{-}/x_{i0}$ is the average redundancy of input, and $1+\frac{1}{s_{1}+s_{2}}(\sum_{i=1}^{s_{1}}s_{r}^{g}/y_{r0}^{g}+\sum_{i=1}^{s_{2}}s_{r}^{b}/z_{r0}^{g})$ represents the efficiency of outputs.

#### 2.2.2. Global Malmquist–Luenberger (GML) Index

In consideration of the continuity and sustainability of urban production activities and variations of technology, the global production technology is combined with ML index to propose the GML (global Malmquist-Luenberger) index model [43], which remedies the defects of non-transitivity and no solution of traditional ML index and achieves inter-temporal comparison. Therefore, this paper adopts the GML index to measure the urban GSDL. Assuming that *N* factors $x=(x_{1},x_{2},\ldots \ldots ,x_{N})\in R_{N}^{+}$ are adopted in each DMU, then *M* desired outputs $y=(y_{1},y_{2},\ldots \ldots ,y_{M})\in R_{M}^{+}$ and *I* undesired outputs $u=(u_{1},u_{2},\ldots \ldots ,u_{I})\in R_{I}^{+}$ are obtained. As a result, the GSDL of cities can be obtained. Defining the GML index is from *t* to *t* + 1, and the according formula is as follows:

(4) $$\begin{array}{l} GML^{t,t+\;1}(x^{t},y^{t},b^{t},x^{t+\;1},y^{t+\;1},b^{t+\;1})\\ =\frac{1+D^{G}(x^{t},y^{t},b^{t})}{1+D^{G}(x^{t+\;1},y^{t+\;1},b^{t+\;1})}\times \frac{1+D^{t}(x^{t},y^{t},b^{t})}{1+D^{t+\;1}(x^{t+\;1},y^{t+\;1},b^{t+\;1})}\\ \times \frac{1+D^{G}(x^{t},y^{t},b^{t})}{1+D^{t}(x^{t},y^{t},b^{t})}\times \frac{1+D^{t+\;1}(x^{t+\;1},y^{t+\;1},b^{t+\;1})}{1+D^{G}(x^{t+\;1},y^{t+\;1},b^{t+\;1})}\\ =EC\times TC \end{array}$$

where $D^{G}(x,y,b)=max\{\beta |y+\beta y,b-\beta b\in P^{G}(X)\}$ represents the global directional distance function of $P^{G}(X)$, which is determined by the global production possibility. The index $GML^{t,t+1}$ represents the variation of GSDL in the decision-making unit from the period of *t* to *t* + 1, and $GML^{t,t+1}>1$ indicates that the urban GSDL of period *t* + 1 has increased since the period of *t*. Assuming that ${GSDL}_{2005}=1$, then the corresponding formula is as follows:

(5) $${GSDL}_{2006}={GSDL}_{2005}\times {GML}_{2006}$$

#### 2.2.3. The Selection of Input and Output Indicators

Based on the previous studies [12,44], this paper adopted the SBM model and GML index to evaluate the urban GSDL with the following indicators shown in Table 1.

In addition, the fixed capital stock was measured by perpetual inventory method [45]. Regional GDP was represented by the real GDP, which took the year of 2005 as the base period. The entropy value method with time variable was adopted to calculate the undesirable outputs.

### 2.3. Empirical Model

#### 2.3.1. Basic Regression Model

To explore the effects of the influencing factors on the urban GSDL, this paper first constructed a quantile regression model to test the constraints of each influencing factor on the urban GSDL. According to the research objectives, the basic panel data regression model was constructed as follow:

(6) $$\begin{array}{l} {GSDL}_{it}=\beta_{0}+\beta_{1}{STR}_{it}+\beta_{2}{LnOPEN}_{it}+\beta_{3}{LnPGDP}_{it}+\beta_{4}{HC}_{it}\\ +\beta_{5}{FS}_{it}+\beta_{6}{SCALE}_{it}+\beta_{7}{GOVERN}_{it}+\varepsilon_{it} \end{array}$$

where the *GSDL_it_* represents the explained variable, and *ε* *_it_* is the random disturbance term. The *i* and *t* represent the time and the individual virtual variables, respectively.

#### 2.3.2. Threshold Model

Equation (6) only expresses the impact of various variables on the urban GSDL. It does not consider that the explanatory variables may have stage changes or be affected by other mechanisms, which is unable to depict the breakpoints of variation in the relationships among explained variables and explanatory variables. Thus, the threshold regression model was constructed to explore the threshold effect of economic and financial development level on the industry structure and opening-up level. Then the basic threshold regression model based on Hansen’s panel regression theory was adopted as follow:

(7) $${GSDL}_{it}=u_{i}+\beta_{1}X_{it}\cdot 1(q_{it}\leq \lambda )+\beta_{2}X_{it}\cdot 1(q_{it}>\lambda )+\varepsilon_{it}$$

where the *GSDL_it_* represents the explained variable, *X_it_* is on behalf of the explanatory variable, *q_it_* is the threshold variable, *γ* is the threshold value to be estimated, and *ε* *_it_* is the random disturbance term. On this basis, the double threshold regression model is constructed as follow:

(8) $${GSDL}_{it}=u_{i}+\beta_{1}X_{it}\cdot 1(q_{it}\leq \gamma_{1})+\beta_{2}X_{it}\cdot 1(\gamma_{1}<q_{it}\leq \gamma_{2})+\beta_{3}X_{it}\cdot 1(q_{it}>\gamma_{2})+\varepsilon_{it}$$

where the threshold value meets the requirement of *γ*_1_ < *γ*_2_, and other variables are the same as Equation (7). The multiple-threshold model can be deduced by analogy.

### 2.4. Index Selection

Explained variable: the Urban GSDL, which is evaluated by the SBM-GML index.

Explanatory variables: (1) Industrial structure (STR), which is measured by the proportion of tertiary industry in GDP. (2) Opening-up level (lnOPEN), which is denoted with the actual utilized foreign investment in the year after exchange rate conversion.

Control variables: (1) Economic development level (lnPGDP). It has been assumed that cities with a higher level of economic development would have more potential to support technological innovation and invest more in environmental governance to improve the urban GSDL. Thus, economic development level was meaningful to be researched in this paper, which was measured by urban per GDP after adjusting for inflation. (2) Human capital level (HC). It was supposed that the increase of human capital was favorable for the accumulation of innovation and technology, which provided talents and technical support for the green and smart development of cities. As such, the human capital level expressed by the number of university students per 10,000 people was adopted in this research [46]. (3) Financial development level (FS). As green finance has become an indispensable supporting method to promote technology innovation of low-carbon emission and green industry development, green and clean industrial production and the coordination of industrial development were further improved. Thus, the financial development level was adopted in this paper to explore the influencing factors of the urban GSDL, which was represented by the ratio of current credit balance to regional GDP [47]. (4) Urban scale (SCALE). The urban scale has been proved to be a symbol of its economic development, which was an essential condition of industrial agglomeration and acceleration of the industrialization process. Thus, this paper adopted the urban scale to control the potential influence of urban GSDL; the urban total population at the end of the year was selected [48]. (5) Government size (GOVERN). It is known that urban sustainable development could not be achieved without the assistance of government policies and funds, while excessive government intervention will also inhibit the development of the market and hinder urban production efficiency. Therefore, the proportion of financial expenditure In the GDP was selected to deepen the research of influencing factors of the urban GSDL [10,49]. The descriptive statistics are shown in Table 2.

## 3. The Spatial-Temporal Evolution Pattern of the Urban GSDL

### 3.1. Temporal Characteristics of the Urban GSDL

**Figure 1:**
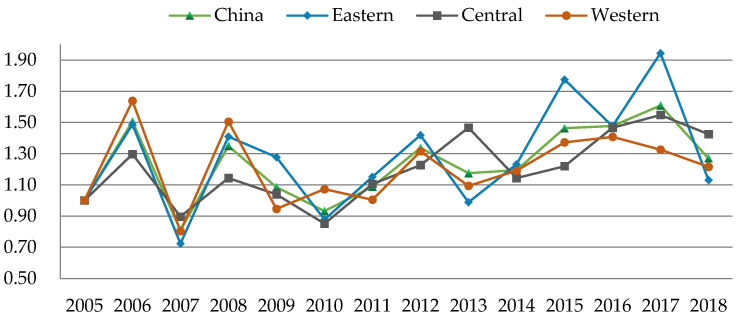
The temporal trend of urban GSDL in China from 2005 to 2018.

Based on the SBM-GML index model, the GSDL of 232 cities in China was evaluated by MAXdea software. The smaller the value is, the lower the urban GSDL is. The overall and regional temporal evolutionary trends of urban GSDL are shown in Figure 1.

As illustrated in Figure 1, the urban GSDL showed a fluctuating growing tendency overall, with an average annual growth rate of 1.935%. Specifically, the urban GSDL could be depicted as an inverted V-shape from 2005 to 2007, and there was a short rise in 2006. This is mainly due to the fact that China’s energy-intensive industries developed fast for achieving high GDP, which caused large amounts of pollution. Since 2005, the outbreak of water pollution in Songhua River and other pollution incidents has spurred ecological awareness in China, thus the government’s environmental regulation has been strengthened. Then, the urban GSDL increased relatively steadily from 2008 to 2013. Furtherly, with the establishment of pollution emission reduction targets during the 11th and 12th Five-Year plans of China, the urban GSDL tended to be promoted stably, thus the negative impact of industrial development has been offset to some extent. The urban GSDL began to accelerate comparatively faster since 2014, which could be mainly attributed to the reinforcement of environmental policies, energy conservation, emission reduction, and the combination of environmental factors in local assessment after the year 2013 [50]. In addition, due to the implementation of low-carbon pilot cities and smart pilot cities projects from 2010 and 2012, the industrial structure was further optimized and upgraded, the efficiency of urban governance was promoted, and the incentives from technological innovation were fully released, which provided a solid foundation to the enhancement of urban GSDL.

From the regional dimension, the urban GSDL was highest in the eastern region, followed by the western and central regions, and the differentiation of the urban GSDL among regions showed no converge. The reason is that most cities in the eastern region may have a higher level of green development, scientific and technological innovation, and governmental governance, which would further promote the urban GSDL. However, cities in the central region are vital basements of energy and raw material, which also undertake domestic and foreign industries, thus their environmental carrying capacity would be reduced [51]. Moreover, the location, transportation accessibility, and information infrastructure construction in the central and western regions are inferior to eastern cities, which restricts the urban GSDL in the central and western regions.

### 3.2. Distribution Characteristics of the Urban GSDL in Different Level

**Figure 2:**
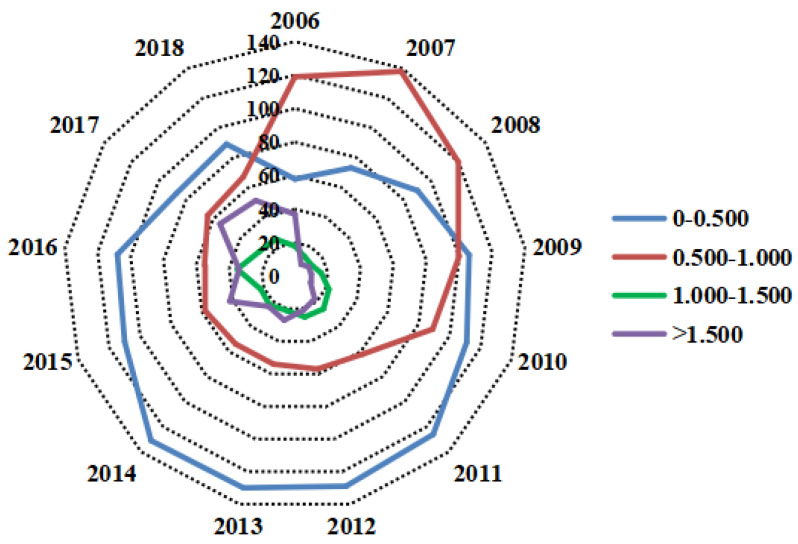
The Distribution characteristics of the urban GSDL in China from 2006 to 2018.

To see the differences of urban GSDL between cities more clearly, the GSDL level was divided into four stages, and cities at different levels were calculated according to the year. Considering urban GSDL of the basic period is the same, so the year of 2005 is not included in the following figure. Specific results are shown in Figure 2.

As can be seen from Figure 2, cities with urban GSDL between (0–0.500) and (0.500–1.000) always counted for a large percent; on the contrary, the urban GSDL belonging to (1–1.500) was less than 40 from 2006 to 2018. It indicated that, although the urban GSDL has been promoted in the past decades, considerate efforts were still required to achieve mutual development. Moreover, cities with high-level GSDL of more than 1.500 increased rapidly after 2014, which was in line with the trend of GSDL in Figure 1. These changes can be largely attributed to the increasingly enhanced environmental regulation, upgraded industry structure, and the pilot cities that focus on the green transformation and smart construction [34,51].

**Figure 3:**
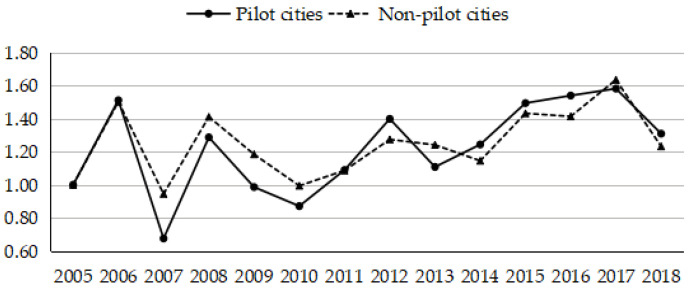
Time trends of urban GSDL between pilot cities and non-pilot cities.

Low-carbon cities and smart cities are concrete practices for China’s sustainable development, which successively launched in the year 2010 and 2012, thus the construction of green and smart cities began. By the end of 2021, China’s low-carbon pilot city has reached 97 provinces and cities; smart cities have involved 95% of China’s deputy provincial cities and 83% of prefecture-level cities. Consequently, this article further explored the differences of urban GSDL between pilot cities and non-pilot cities. The results are shown in Figure 3.

As can be seen from Figure 3, the pilot cities and the non-pilot cities had a similar fluctuation trend before 2011, and the GSDL of the pilot cities was relatively low. After the year 2011, the gap between pilot cities and non-pilot cities became gradually narrowed, and the GSDL of the pilot cities was higher than non-pilot cities on the whole. The results indicate that the objectives of the low-carbon pilot policy and smart city policy are consistent with the construction of green and smart cities. This is mainly due to the fact that the construction of green and smart cities embody intelligence and environmental protection conceptions, which make full use of technology innovation to solve the bottleneck of urban development. On the one hand, with the improvement and deepening of the low-carbon pilot cities, new environmental policies such as financial and credit, securities, and funds have been widely adopted in the process of green and low-carbon transformation. On the other hand, China has issued more than 100 documents for guidance at the national level since the concept of smart city was put forward, involving supply, environmental, and demand policies. Due to the policies related to low-carbon pilot cities and smart cities the industrial structure and the intelligence services of urban infrastructures could be optimized and upgraded, which in turn promotes the urban GSDL.

### 3.3. Spatial Characteristics of the Urban GSDL

**Figure 4:**
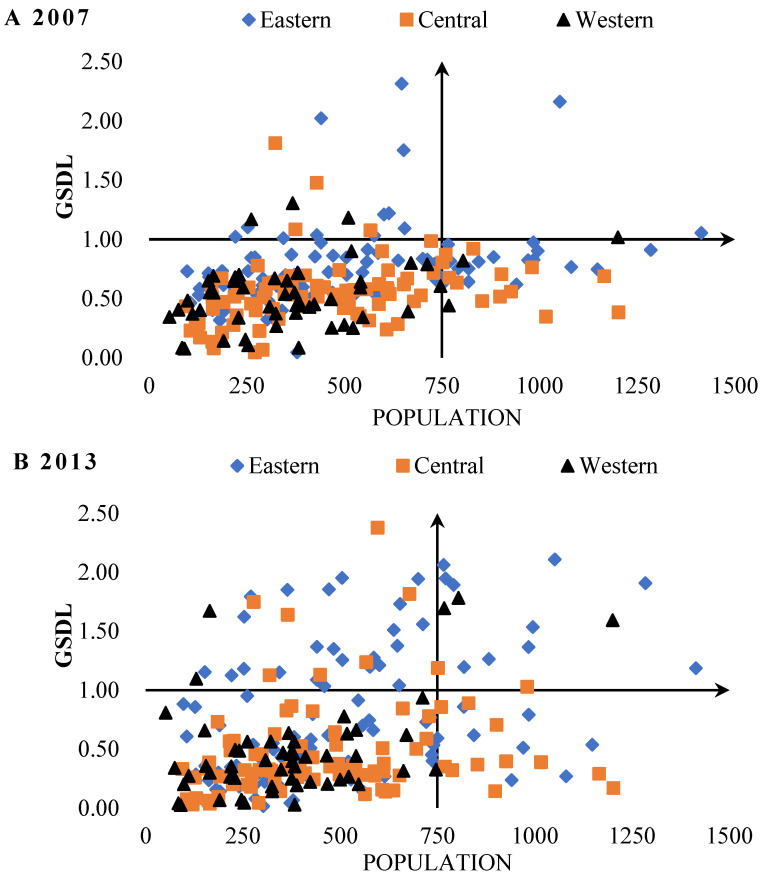

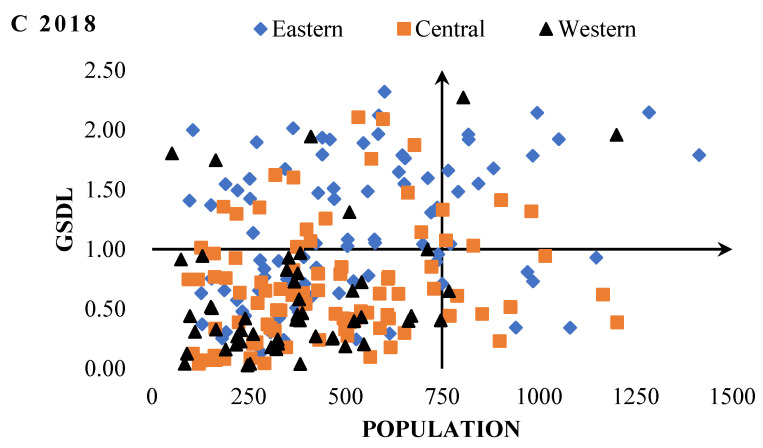
The spatial trend of the urban GSDL (Green and Smart Development Level) in China in the year of 2007 (**A**), 2013 (**B**), and 2018 (**C**) respectively.

To explore the characteristics of spatial changes among different regions, this paper divided the research period into three stages according to the study period span, and the time nodes are the years of 2007, 2013, and 2018, respectively. The results are shown in Figure 4.

According to Figure 4, the urban GSDL in eastern and central regions has been improved dramatically from 2007 to 2018, while it was relatively lower in western region, which showed no significant promotion within these years. This is mainly due to the fact that some cities in the eastern region would mostly take reform ahead, execute strict environmental regulations, and have solid innovation foundations, which are beneficial to promote the urban GSDL. In addition, the rapid development of the e-commerce industry has installed a new engine for the economy, and the maturity of artificial intelligence, blockchain, and other technologies have further improved the allocation efficiency of resources and promoted urban environmental governance and economic growth in eastern and central regions. The possible reason for the lower urban GSDL in western regions is that the resource-based cities are mainly aggregated in the west. The heavy industry structure, shortage of talents, and mismatch between energy supply and demand may lead to the dilemma of “Resource Curse” and “Bottom Line Competition” in the process of development, which inhibits the improvement of urban GSDL in the western region. In addition, the relatively backward development of the economy and investment environment hinders the circulation of resources and elements, as well as the scientific and technological development, which further constrains the development of urban GSDL in the western region.

### 3.4. Efficiency Decomposition of the Urban GSDL

**Figure 5:**
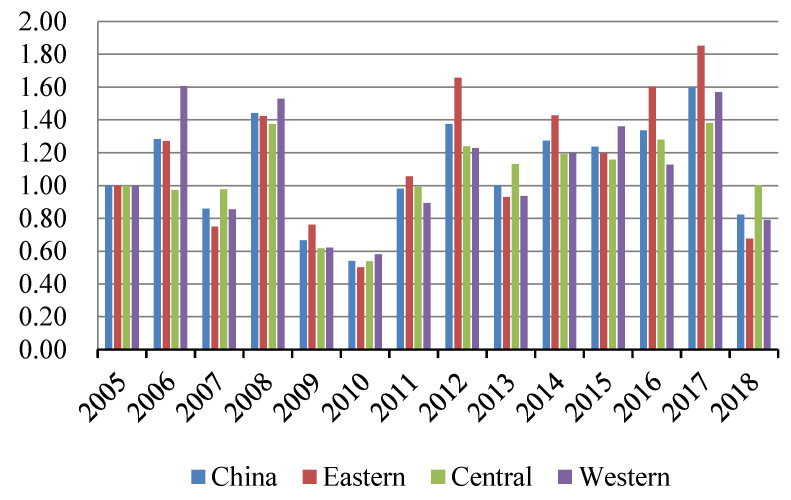
Time trends of technical progress (TC).

**Figure 6:**
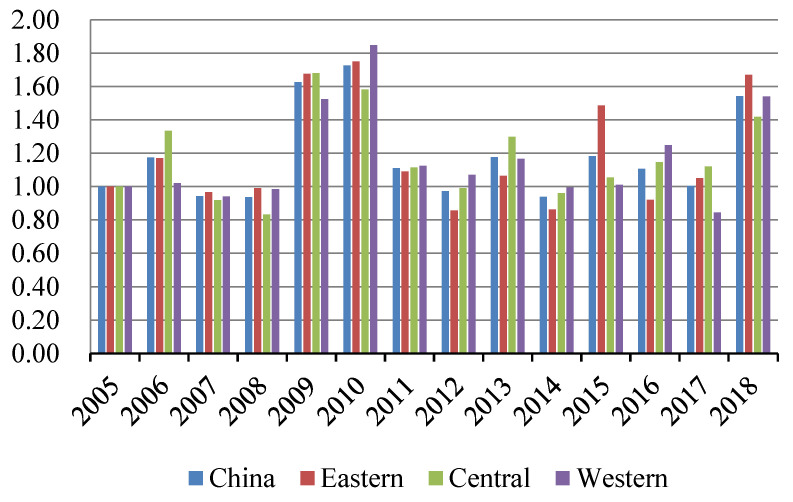
Time trends of technical efficiency (EC).

According to the research of Tone [41], the GML index could be decomposed into technical progress index (TC) and technical efficiency variation index (EC). When the TC > 1, it indicates that the expected outputs of the urban GSDL increase, while the unexpected output decrease. On the contrary, the production frontier of the cities is inverted. When the EC > 1, it indicates the technical efficiency of the urban GSDL is improved compared with the previous period. The technical efficiency decomposition results of the urban GSDL are shown in Figure 5 and Figure 6.

As shown in Figure 5 and Figure 6, the average technical efficiency and technical progress of the urban GSDL from 2005 to 2018 presented a fluctuating “Two-Wheel-Drive” effect on the urban GSDL. The contribution of technical progress was greater than that of technology efficiency from 2005 to 2008, and the technology efficiency was improved significantly from 2009 to 2010. It was worth noting that the average technical efficiency and technical progress changed stably after 2011, and the fluctuating “Two-Wheel-Drive” effect was more obvious. From the perspective of the regional dimension, the technical progress and technical efficiency of the three regions fluctuated obviously from 2005 to 2011. The possible reason was that the economic crisis in 2008 has brought a series of sequelae to China, as well as the immature science and technology industry and the heavy industry structure. Furtherly, natural disasters have also restricted production efficiency. The differentiation degree of the technical efficiency and technical progress has increased among regions since 2012 and the contribution of technical progress on the urban GSDL became more significant, while the driving force of technical efficiency weakened a little bit. This may be due to the low-carbon pilot cities and smart cities construction policies launched in 2010 and 2012, successively, which further strengthened the green and smart infrastructure constructions. As such, the innovation compensation, and the effect of scientific management on the urban development were further accumulated, and the allocation efficiency of resource elements was effectively consolidated, which all promoted the development of urban GSDL dramatically.

## 4. Analysis of Influencing Factors of the Urban GSDL

### 4.1. Theoretical Mechanism Analysis

**Figure 7:**
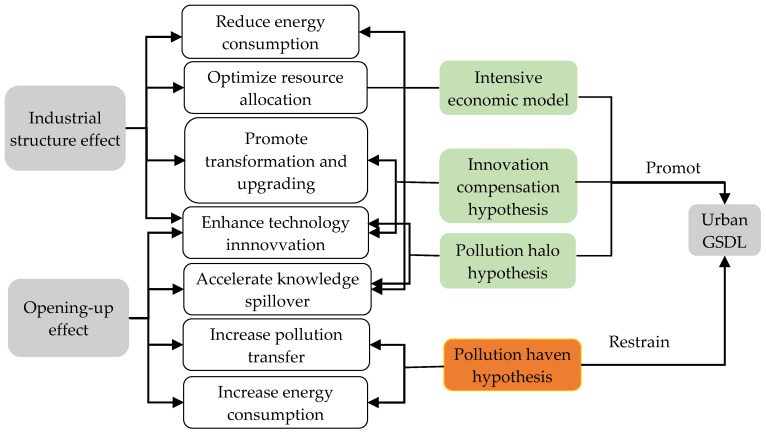
Theoretical mechanism diagram of influencing factors on the urban GSDL.

The construction of green and smart cities is a complex system, which would be influenced by muti-factors and conversely affects urban development. Based on the current literature, this paper adopted variables from the two perspectives of industrial structure and opening-up to explore their effects on the urban GSDL. The specific mechanism analysis can be seen in Figure 7.

#### 4.1.1. Mechanism Analysis of Industrial Structure to the Urban GSDL

Industrial structure effect. It has been proved that industrial structure is the major influencing factor of urban sustainable development [52]. Industrial structure can possibly exert influence on the urban GSDL through optimizing resource allocation, promoting transformation, and upgrading of industrial structure [53,54]. On the one hand, industrial energy consumption and emission in China is the main source of environmental pollution. In general, the lower the proportion of the secondary industry is, the higher the urban GTFP is. To achieve sustainable development, China has launched numerous policies to promote the transfer of resources to high-value-added industries and reduce fossil energy consumption, which is conducive to achieving an intensive development model and reducing pollution. On the other hand, technology innovation could enhance the positive impacts on the urban GSDL. Urban smart construction can be promoted through technology innovation of tertiary industry [53]. With the construction of green and smart development, cities are more prone to promote technological innovation and make efforts to accelerate the transformation of growth drivers and industrial structure adjustment, which contribute to the transformation from extensive industry to intensive industry. Additionally, the development of high-tech industries and emerging industries would make the elements flow to the industries which are more in accordance with the notion of sustainable development and realize the optimization and upgrading of industrial structure. It has been demonstrated that the rationalization and the upgrading of industrial structure can promote urban green and smart efficiency [54]. Therefore, the structural effect on green and smart development will have a significant role in promoting urban GSDL.

#### 4.1.2. Mechanism Analysis of Opening-Up Level to the Urban GSDL

Opening-up effect. Research has proved that continuous expanded opening-up level to overseas has two-sided impacts on China’s environment and economy. The “pollution haven hypothesis” and the “pollution halo hypothesis” provide a theoretical basis for the negative and positive effects of opening-up on the urban green transformation and the construction of smart cities, respectively [55]. On the one hand, an open economy makes contributions to the introduction of advanced technologies and talents to local companies, as well as the knowledge spillover effects that would be further released. Consequently, the urban GSDL can be promoted through technology innovation and upgrading of productive elements [56], which is denoted as the “pollution halo hypothesis”. Nevertheless, the local governments in China may usually lower environmental standards to attract foreign direct investment [57], which leads to environmental pollution problems such as carbon emissions. Thus the “pollution haven hypothesis” could be supported. Furthermore, unreasonable foreign investments might also aggravate domestic environmental pressure by transferring high-polluting industries to host countries and increasing energy consumption, which ultimately hinders the promotion of urban GSDL [35].

### 4.2. Regression Results Analysis

#### 4.2.1. Quantile Regression

Mean regression can only describe the central tendency of conditional distribution effect of X on Y. However, there are many extreme values and heteroscedasticity. To solve these deficiencies, the quantile regression method was put forward [58], which could estimate the coefficients of explanatory variables corresponding to different loci and overcome the effect of extreme values. This paper selected loci of 10%, 25%, 50%, 75%, and 90%, respectively, and adopted the bootstrap method to estimate the influencing factors of urban GSDL. To compare the differences between mean regression and quantile regression, the results are described in Table 3.

As shown in Table 3, there was a significant and positive effect of industry structure on the urban GSDL. With the increase of the loci, the promoting effect of industry structure on the urban GSDL increased gradually. This could be attributed to the constantly improved industry structure, as such the allocation efficiency of production resources was further optimized, and the production efficiency was improved as well. Additionally, the coefficient of lnOPEN showed the “W” pattern, which was always significant and positive, indicating that opening-up showed no “Pollution Paradise” in this scenario. On the contrary, lnOPEN showed a negative effect with mean regression. As research showed that the positive effect of opening up on the development of the green economy was not stable, there was a game between positive and negative effects [59]. In fact, cities with low technology and a low-quality supply of goods would seek international trade with cheaper labor and easier access to resources and the environment. While the potential negative effects of international trade on resources and the environment are time-delayed, the economic compensation of international trade would also amplify its positive impact. In contrast, there are thorough environmental regulations and international trade systems in cities of high-level opening-up, and their development model of “environment–economy–society” could be mature. As such, the negative effects can be “filtered” to some extent, and the opening-up could play a more stable role in promoting the green and smart development of cities [60]. In terms of the control variables, the economic development level inhibited the urban GSDL overall. The possible reason is that the urban economic development model, which often sacrifices environmental sustainability, needs to be optimized. In addition, there was no significant effect of FS on urban GSDL at the lower level, while it became significant with the increasing of the loci. The reason is mainly due to the improvement of the financial market, which could heighten the risk dispersion ability of cities, stimulate technological innovation, and promote smart infrastructure construction. As a consequence, the green and smart development of cities would be promoted ultimately [61,62]. However, the influence of human capital level on the urban GSDL varied from insignificant to negative with the increasing of the loci. This might be because cities with higher GSDL have solid economic foundations, and relatively abundant financial conditions, which would be a benefit to high-quality talents attraction. However, the imbalanced structure between talents supplies and urban demand inhibits the role of human capital in improving the urban GSDL. The urban scale showed a significant but limited positive effect on the urban GSDL, indicating that excessive urban scale would lower the urban GSDL potentially. In other words, the reasonable expansion of the urban scale would accelerate the urbanization process, improve production intensification, and improve pollution governance. However, the excessive urban scale might increase the difficulty of governmental governance and stimulate energy consumption, as well as pollution emissions generated in the production and living process, which consequently hinders the transformation and upgrading of green production. Moreover, it was shown that the government size inhibited the urban GSDL, which might be related to the structure of fiscal expenditure. The excessive investment of government in the production field would squeeze the investment for R&D of green energy and environmental governance, thus inhibiting the growth of urban green-smart efficiency. In addition, excessive government intervention tends to reduce the conversion rate of scientific and technological achievements, which would impede the green technology innovation and the construction of smart application platforms of cities.

#### 4.2.2. Difference Analysis

Due to the differences in the development basis and speed of regional cities and the heterogeneity of influencing factors among regions, this paper further divided samples into three groups to explore the differentiation characteristics based on China’s administrative division, eastern, central, and western. The results are shown in column (2) to column (4) of Table 4. Then, the heterogeneous influence of low-carbon pilot cities and smart pilot cities of urban GSDL were elaborated. The policy virtual variables are set, where Treat1 represents the low-carbon pilot policy and Treat2 represents the smart pilot policy. The value of all cities meets the condition of Treat1 = Treat2 = 0 before policy implementation, while after the implementation of the pilot policy, the value of pilot cities meets the condition of Treat1 = Treat2 = 1, otherwise Treat1 = Treat2 = 0. Adding Treat1 and Treat2 into the regression model (6). The results are shown in column (5) of Table 4.

As described in Table 4, industrial structure and the urban scale showed positive effects of indifferent degrees on the urban GSDL in eastern and western regions. While economic development level showed a negative effect on the urban GSDL in the central and western regions, in which the coefficients were −0.3109 and −0.4056, respectively. Nevertheless, the negative effects of government size on the urban GSDL were mainly embodied in the eastern and central regions, and the coefficients were −1.4362 and −0.8303, respectively. The financial development level only promoted the urban GSDL of the central region, and the positive effect of human capital was insignificant. The results further suggested that cities in different regions should take measures according to their development and geographical conditions and take corresponding actions to release the positive potential of influential factors, reduce the negative impact of factors. Column (5) of Table 4 furtherly revealed that the low-carbon and smart pilot policies have effectively promoted the urban GSDL, which was consistent with the findings in Figure 3.

### 4.3. Threshold Effects

Threshold test can not only explore the relationship among variables, but also can depict breakpoint of relationship. As China is a country with huge developing differences among cities, as consequence, cities may also need to reach a threshold to achieve long-term construction of green and smart cities. Specifically, cities with high economic development level will have greater support for technological innovation, and more investment for pro-environmental issues and infrastructure construction, thus the urban GSDL would be enhanced. Furtherly, cities with a low level of financial development are more likely to lack risk dispersion capabilities, and the developed financial market is conducive to the long-term stability of technology upgrading behavior, to improve the total factor productivity [63,64]. Thus, the threshold effect of economic and financial development level on the industry structure and opening-up level were explored for further analysis, which could reflect urban characteristics to some extent. The regression results are shown in Table 5. By comparing the threshold effects of the economic development level and financial development level on industrial structure and opening-up level, it was shown that the industrial structure played a stronger role in promoting the urban GSDL under the constraint of economic development level, and the opening-up level promoted the urban GSDL more effectively under the constraint of the financial development level. As shown in Table 5, in the scenario of single threshold, when the economic development level was on the left side of the threshold (lnPGDP < 11.4022), the effect of industrial structure on the urban GSDL was insignificant, while when lnPGDP > 11.4022 their effect became significant at 1% level. In the scenario of the double threshold, when the economic development level was over the second threshold (lnPGDP > 11.5857), the effect of the industrial structure on the urban GSDL was significantly at 1% level with the coefficient of 0.5679. By contrast, when the financial development level was over the single threshold (0.9900), the positive impact of the opening-up level on the urban GSDL increased from 0.0381 to 0.0848, and the positive effect increased to 1.1072 when the financial development level was on the right side of the second threshold (1.3100). This may be due to the fact that a city with a higher financial development level generally takes less risk of production efficiency in the trade sector, which contributes to promoting the urban GSDL indirectly. Furtherly, the high level of opening-up is significantly related to the mature financial market, which provides lots of opportunities for international capital and talent to flow into the domestic market, thus improving China’s technical ability, optimizing the allocation of elements, and improving the quality of urban development. As a result, the financial development level could enhance the driving role of the opening-up on the urban GSDL [65].

## 5. Conclusions and Implications

### 5.1. Conclusions

Under the background of green and smart cities construction in China, it is urgent to figure out what are the crucial influencing factors of the urban GSDL to break through the shackles of the unhealthy economic development model. This paper developed the GTFP by adopting smart input-output elements and evaluated the urban GSDL of 232 cities in China from 2005 to 2018 based on the SBM-GML model. Then the influencing factors of the urban GSDL were tested with quantile regressions and threshold regressions.

The main research conclusions are as follows:

From the perspective of spatial-temporal evaluation characteristics of urban GSDL, China’s urban GSDL increased overall and showed a fluctuating growth trend overall from 2005 to 2018. The technical progress and technical efficiency showed unlike positive effects on the urban GSDL at different stages, which could be characterized as the “Two-Wheel Drive” effect.

Concerning the influencing factors, it can be observed that the positive effect of industry structure was enhanced with the increasing of the loci, and the opening-up level promoted the urban GSDL overall, which did not confirm the “Pollution Refuge Hypothesis” as previous research [66]. In terms of other influencing factors, the urban scale showed a positive impact on the urban GSDL overall, while the restraining effects of economic development level and the government size on the urban GSDL were obvious. With the increase of the loci, the driving role of financial development level on the urban GSDL was enhanced gradually, while the influence of human capital level on the urban GSDL changed from insignificant to negative, which was not in line with the expectations.

By constructing the threshold regression model, it was shown that when the economic and financial development level exceeded a particular threshold, the industrial structure and opening-up level could promote the urban GSDL more effectively. In particular, the single threshold effect of economic development level and financial development level on the industrial structure was significant, but there was no double threshold effect of financial development level on the industrial structure. Moreover, both single and double threshold effects of economic and financial development on the opening-up level were significant and positive, while the threshold effect of economic development level on the industry structure was distinct overall. By contrast, the threshold effect of financial development level on the opening-up level was more significant compared with the economic development level.

### 5.2. Policy Implications

Based on the above analysis, corresponding suggestions are put forward from the following three aspects to promote the urban GSDL in the next stage:

Firstly, the governments are supposed to play full potential of the leading cities. Moreover, it is necessary to highlight the leading and exemplary role of cities with a higher level of GSDL and weaken the “Glass Wall” barriers between cities. In addition, backward cities must catch up to the frontier cities and make great efforts to foster a social environment for green and smart development and a regional collaborative innovation system. Meanwhile, they are supposed to enhance the learning and absorption capacity and accelerate the growth of urban GSDL, which is meaningful to grasping the opportunities of city groups development, such as Beijing-Tianjin-Hebei, Yangtze River Delta, and the Guangdong-Hong Kong-Macao Greater Bay Area. Furthermore, a new collaborative development model of “Central cities leading urban agglomerations and promoting surrounding regions” should be established to improve the urban GSDL.

Secondly, the governments are obliged to enhance the supporting role of science and technology in improving the smart innovation of cities. On the one hand, urban innovation proceeded based on the national strategy is the premise of accelerating basic research and key technologies, which provides solid foundations to ensure the initiative of innovation players in personnel training, intellectual property protection, and innovation platform incubation. On the other hand, it is of great importance to give full play of the strategic and intelligent support of talents, guarantee the working conditions, innovation performance, and achievement transformation of scientific and technological workers with a systematic and comprehensive system of “Introduction-Cultivate-Training”.

Thirdly, cities must make efforts to construct a coordinated development system and optimize the efficiency of resources allocation. Furthermore, it is necessary to break the barriers of inter-regional talents flow, promote the sharing of human capital in the process of urban agglomeration and regional integration [67]. Additionally, cities should take pages from previous experience to break the current dilemma of ineffectiveness or inhibiting impact of human capital on urban GSDL and realize the rational disposition of different types of talents between cities. What calls for special attention is that governments should pay attention to introducing effective capital, elements, as well as resources of production continuously and optimize the structure of the foreign investment through the high-quality opening-up. Consequently, cities could make a more concentrated and efficient investment in strategic industries and projects with comparative advantages. Besides, controlling the urban scale and the government size appropriately is necessary for cities as well as governments. The establishment of an urban development system where the government plays a dominating role, the market plays a leading role, and multiple participators are included is also crucial to promote the urban GSDL. In consequence, more funds will be distributed to clean energy, environmental governance, and the social and private capital will flow into smart infrastructures such as energy, transportation, and information. As a result, the urban GSDL would be improved ultimately.

This study focused on the spatial and temporal characteristics and the influencing factors of urban GSDL. By attempting to explore the evolution characteristics with more consideration of smart factors and elaborating the influence mechanism of industrial structure and opening-up effects, this research extended the study on traditional quality evaluation of urban development. Therefore, the main contribution of this project was expanding the temporal and spatial evolutionary heterogeneity of the urban GSDL in China by adopting a more comprehensive measurement including green and smart factors as input and output elements. This paper also explored the influencing factors of urban GSDL from multi-dimensions with quantile test and threshold test. However, the empirical model constructed in this paper to investigate the factors affecting the urban GSDL can be extended and applied from multiple perspectives. Spatial agglomeration could be further explored in future research. In addition, indicators that represent smart factors need a more detailed and comprehensive investigation. This is what we need to improve and strengthen in future research.

## Figures and Tables

**Table 1:** Construction of input and output indicators.

Indicator	Variable	Unit	Computation Method
Input indicators	Fixed capital stock	100 million yuan	Perpetual inventory method
	Labor	10 thousand people	The number of urban employees at the end of year
	Electricity consumption	10 thousand kilowatts	Total electricity consumption
	Education and technology expenditure	10 thousand yuan	Financial expenditure on science, technology, and education
Output indicators	Regional GDP	100 million yuan	Regional GDP of the year
	Books collected in public libraries	Ten thousand volumes	The number of urban books in public libraries
	Patent application quantity	Part	The number of urban patent application
	Discharge of industrial waste water	10 thousand tons	Industrial waste water discharge volume of the city
	Industrial smoke and dust emissions	Tons	Industrial smoke and dust emissions’ volume of the city
	Industrial SO2 emissions	Tons	Industrial SO2 emissions’ volume of the city

**Table 2:** Descriptive statistics of variables.

Variables	Mean	Std. Dev	Min	Max
GSDL	0.8933	0.9929	0.0282	2.2144
STR	38.83	9.50	16.99	77.37
lnOPEN	11.93	1.80	5.58	16.04
lnPGDP	10.39	0.74	8.35	12.46
HC	18.61	23.75	0.32	122.65
FS	0.85	0.50	0.25	3.86
SCALE	469.21	269.34	72.30	1456.00
GOVERN	0.16	0.09	0.02	1.17
Observation	3248	3248	3248	3248

**Table 3:** Quantile regression results.

Variables	Q = 0.10	Q = 0.25	Q = 0.5	Q = 0.75	Q = 0.90	Mean Regression
STR	0.0025 ** (0.0012)	0.0052 *** (0.0010)	0.0070 (0.0017)	0.0138 *** (0.0027)	0.0228 *** (0.0066)	0.0229 *** (0.0027)
lnOPEN	0.0254 *** (0.0060)	0.0522 *** (0.0057)	0.0734 *** (0.0078)	0.0668 *** (0.0136)	0.0809 *** (0.0284)	−0.0487 * (0.0190)
lnPGDP	−0.0435 *** (0.0141)	−0.0476 *** (0.0135)	−0.0519 *** (0.0187)	−0.0553 ** (0.0229)	0.0960 (0.0595)	−0.3109 *** (0.0406)
HC	0.0002 (0.0008)	0.0005 (0.0005)	−0.0012 * (0.0006)	−0.0045 *** (0.0013)	−0.0131 *** (0.0023)	0.2356 *** (0.0678)
FS	0.0057 (0.0211)	0.0369 (0.0238)	0.1305 *** (0.0398)	0.4101 *** (0.0913)	0.7288 *** (0.2046)	0.0014 *** (0.0028)
SCALE	0.0002 *** (0.00003)	0.0002 *** (0.00003)	0.0002 *** (0.00004)	0.0023 *** (0.0001)	0.0009* (0.0002)	0.0046 *** (0.0005)
GOVERN	−0.2758 ** (0.0225)	−0.0049 *** (0.0019)	−0.0024 (0.0018)	−0.0036 *** (0.0405)	−0.0055 *** (0.0006)	−0.8303 *** (0.2458)
Cons	0.7162 ** (0.1355)	0.0173 (0.1455)	0.1033 (0.2033)	−0.1826 ** (0.2553)	−1.7957 *** (0.5332)	1.5466 *** (0.3394)
Samples	3248	3248	3248	3248	3248	3248

Note: The ***, **, * indicating significance at 1%, 5% and 10%, respectively.

**Table 4:** Analysis of the differences in the influencing factors.

Variables	Eastern	Middle	Western	China
STR	0.0407 *** (0.0085)	0.0229 *** (0.0027)	0.0153 *** (0.0039)	0.0190 *** (0.0027)
lnOPEN	−0.1148 ** (0.0553)	−0.0487 ** (0.0190)	−0.0664 *** (0.0247)	−0.0537 *** (0.0187)
lnPGDP	0.0623 (0.1269)	−0.3109 *** (0.0406)	−0.4056 *** (0.0497)	−0.3492 *** (0.0407)
HC	0.0042 (0.0103)	0.0014 (0.0028)	0.0006 (0.0044)	−0.0022 (0.0028)
FS	−0.0602 (0.1763)	0.2356 *** (0.0678)	0.1031 (0.1049)	0.1757 *** (0.0670)
SCALE	0.0094 *** (0.0018)	0.0046 *** (0.0005)	0.0044 *** (0.0009)	0.0038 *** (0.0005)
GOVERN	−1.4362 ** (0.6191)	−0.8303 *** (0.2458)	−0.5144 (0.3634)	−0.9834 *** (0.2148)
Treat1				0.1333 *** (0.0515)
Treat2				0.6946 *** (0.0666)
Cons	−4.2647 *** (1.0661)	1.5466 *** (0.3394)	2.9825 *** (0.4557)	2.6072 *** (0.3560)

Note: The ***, ** indicating significance at 1%, and 5% respectively.

**Table 5:** Threshold effect regression.

		lnPGDP		FS	
		Single Threshold	Double Threshold	Single Threshold	Double Threshold
STR	Threshold value	11.4022	9.0153	0.9900	0.6600
			11.5857		0.9900
	F value	112.81 ***	953.75 ***	78.03 ***	70.11 ***
	*p* value	0.0000	0.0320	0.000	0.6520
	Minimum residual sum of square	0.0556	0.7028	0.0020	0.0032
	(q ≦ γ1)	0.0034 (0.0024)	0 (omitted)	0.0041 (0.0025)	0.0009 (0.0027)
	(q > γ1/γ1 < q ≦ γ2)	0.0286 *** (0.0026)	0.5214 *** (0.0096)	0.0154 *** (0.0023)	0.0048 *** (0.0026)
	(q > γ2)		0.5679 *** (0.0127)		0.0147 *** (0.0023)
lnOPEN	Threshold value	11.4002	11.4022	0.9900	0.9900
			11.5167		0.9900
			10.9710		1.3100
	F value	113.19 ***	111.36 ***	83.30 ***	76.11 ***
	*p* value	0.0000	0.0000	0.0000	0.0000
	Minimum residual sum of square	0.0516	0.0615	0.0035	0.0026
	(q ≦ γ1)	0.0686 *** (0.0111)	0.0436 *** (0.0114)	0.0381 *** (0.0132)	0.0447 *** (0.0132)
	(q > γ1/γ1 < q ≦ γ2)	0.1576 *** (0.0110)	0.0807 *** (0.0109)	0.0848 *** (0.0137)	0.0808 *** (0.0137)
	(q > γ2)		0.1593 *** (0.0111)		0.1072 *** (0.0145)

Note: bootstrap is 1000 times, *** indicating significance at 1%.

## Data Availability

Not applicable.
